# Glutathione Redox Changes in Pulmonary Arterial Blood After Remote Ischemic Conditioning in Pulmonary Hypertension: A Single-Arm Pilot Study

**DOI:** 10.3390/antiox15091203

**Published:** 2026-09-19

**Authors:** Suqiao Yang, Linji Li, Junwei Zhang, Xinhua Qiao, Weiyan Sun, Wenbo Zhao, Huimin Hu, Qiumeng Xi, Yidan Li, Xiaojuan Guo, Jianfeng Wang, Juanni Gong, Yuanhua Yang, Chang Chen, Xunming Ji, Xu Zhang, Qi Yang

**Affiliations:** 1 Department of Respiratory and Critical Care Medicine, Beijing Institute of Respiratory Medicine, Beijing Chao-Yang Hospital, Capital Medical University, Beijing 100020, China; yangsuqiao@126.com (S.Y.);; 2 Evidence-Based Medicine Center, Beijing Chao-Yang Hospital, Capital Medical University, Beijing 100020, China; 3 Beijing Key Laboratory of Multimodal Intelligent Diagnosis and Treatment System for Respiratory Diseases, Beijing 100020, China; 4 Department of Radiology, Beijing Chao-Yang Hospital, Capital Medical University, Beijing 100020, China; linji0625@163.com (L.L.);; 5 Institute of Biophysics, Chinese Academy of Sciences, Beijing 100101, China; 6 Department of Physiology and Pathophysiology, Tianjin Medical University, Tianjin 300070, China; 7 Department of Neurology, Xuanwu Hospital, Capital Medical University, Beijing 100053, China; 8 Beijing Key Laboratory of Hypoxic Conditioning Translational Medicine, Xuanwu Hospital, Capital Medical University, Beijing 100053, China; 9 Department of Echocardiography, Beijing Chao-Yang Hospital, Capital Medical University, Beijing 100020, China; 10 Department of Intervention, Beijing Chao-Yang Hospital, Capital Medical University, Beijing 100020, China; 11 China-America Institute of Neurology, Xuanwu Hospital, Capital Medical University, Beijing 100053, China; 12 Beijing Institute for Brain Disorders, Capital Medical University, Beijing 100053, China; 13 State Key Laboratory of Vascular Homeostasis and Remodeling, Department of Biophysics, School of Basic Medical Sciences, Peking University Health Science Center, Beijing 100191, China; 14 Laboratory for Clinical Medicine, Capital Medical University, Beijing 100069, China; 15 Center for Diagnosis and Treatment of Pulmonary Hypertension, Beijing 100020, China

**Keywords:** pulmonary hypertension, remote ischemic conditioning, oxidative stress, glutathione, redox biomarkers, pulmonary arterial blood

## Abstract

Oxidative stress contributes to pulmonary hypertension (PH), but acute redox responses within the pulmonary circulation remain poorly characterized. In this prospective, open-label, single-arm pilot study, 15 patients with PH underwent one 50 min session of remote ischemic conditioning (RIC) during right heart catheterization. Pulmonary arterial (PA) blood was collected immediately before and after RIC. The prespecified primary biomarker family comprised reduced glutathione (GSH) response and the oxidized glutathione-to-GSH (GSSG/GSH) ratio in PA blood-cell and plasma fractions. PA blood-cell GSH response increased (mean paired difference, 118; 95% CI, 83–152; Holm-adjusted *p* < 0.001), whereas the blood-cell GSSG/GSH ratio decreased (−0.64; 95% CI, −0.81 to −0.46; Holm-adjusted *p* < 0.001). Plasma glutathione measures were not significant after multiplicity adjustment. PA lactate, the prespecified secondary endpoint, decreased by 0.45 mmol/L (95% CI, 0.20–0.71; *p* = 0.002). All participants completed RIC without clinically evident acute adverse events. These preliminary findings identify acute PA blood-cell glutathione and metabolic changes associated with RIC; however, the single-arm design precludes causal inference, and confirmation in randomized sham-controlled studies is warranted.

## 1. Introduction

Pulmonary hypertension (PH) is a progressive disorder associated with pulmonary vascular remodeling and right ventricular (RV) dysfunction despite advances in targeted treatment strategies [1,2,3]. Oxidative stress is increasingly implicated in these processes and may reflect disease activity and treatment-associated biological changes [4,5,6,7,8,9].

Among redox regulatory systems, the glutathione pathway is a key intracellular antioxidant defense mechanism [10]. Reduced glutathione (GSH) acts as a major antioxidant buffer, and the oxidized glutathione (GSSG)/GSH ratio reflects the cellular redox state [11,12]. Glutathione-related metabolic alterations have been associated with PH severity and RV remodeling in experimental PH, suggesting potential pathophysiological relevance [6,13]. Clinical studies of oxidative stress in PH have predominantly evaluated circulating biomarkers in peripheral blood or urine, whereas direct assessment of redox changes in pulmonary arterial blood remains limited [14,15].

Remote ischemic conditioning (RIC) is a noninvasive intervention involving repeated cycles of transient limb ischemia and reperfusion. It has been investigated for potential organ-protective effects in cardiovascular and cerebrovascular diseases [16,17]. The underlying mechanisms of RIC are complex and include modulation of oxidative stress, inflammatory signaling, endothelial function, and mitochondrial stress responses, pathways closely linked to PH pathophysiology [18,19]. Among these pathways, modulation of cellular antioxidant defenses may be particularly relevant because glutathione synthesis and recycling are closely linked to redox-responsive signaling pathways, including nuclear factor erythroid 2–related factor 2 (Nrf2) [18]. Experimental studies have shown protective effects of remote ischemic conditioning against pulmonary ischemia–reperfusion injury [20,21,22]. In humans, ischemic conditioning has been reported to blunt hypoxia-induced increases in pulmonary artery pressure and improve oxygenation in healthy volunteers [23,24,25]. Whether similar acute biological responses occur in patients with established PH, particularly within the pulmonary circulation, remains unknown.

In this study, we performed a prospective single-arm biomarker pilot study in patients with PH undergoing right heart catheterization (RHC). By integrating pulmonary arterial (PA), peripheral arterial, and venous blood sampling with separate blood-cell and plasma analyses, we aimed to (1) determine whether a measurable glutathione-related redox signal could be detected in blood sampled directly from the pulmonary artery following RIC, (2) evaluate the short-term tolerability of a single RIC session in patients with PH, and (3) explore accompanying changes in RV function, blood gases, oxygen transport, and hemodynamics as hypothesis-generating end points. These exploratory analyses were intended to generate hypotheses and inform the design of future randomized sham-controlled studies.

## 2. Materials and Methods

### 2.1. Study Design and Study Population

This single-center, prospective, open-label, single-arm biomarker pilot study enrolled 15 patients with PH. The primary objective was to characterize acute changes in PA glutathione-related redox biomarkers following a single session of RIC. Short-term tolerability and accompanying physiological responses were also assessed as exploratory outcomes. The study protocol was approved by the Ethics Committee of Beijing Chao-Yang Hospital, Capital Medical University (approval no. 2025-ke-508), and the study was conducted in accordance with the Declaration of Helsinki. Written informed consent was obtained from all participants. The primary and secondary outcomes were specified in the ethics-approved study protocol before enrollment of the first participant. The study was retrospectively registered in the Chinese Clinical Trial Registry (ChiCTR2600119029) on 14 February 2026, after recruitment had commenced but before enrollment of the final participant, database lock, and outcome analysis. The delay in registration occurred because the investigators were not aware at study initiation that prospective registration was required for this pilot intervention study. These prespecified outcomes were not modified after enrollment began or during outcome analysis.

Eligible participants were adults aged ≥18 years with pulmonary arterial hypertension, chronic thromboembolic pulmonary hypertension (CTEPH), or PH associated with lung disease and/or hypoxia, diagnosed according to the 2022 ESC/ERS Guidelines [2], and who provided written informed consent.

Exclusion criteria were surgically eligible CTEPH; conditions precluding safe application of RIC, including upper-limb thrombosis, deformity, or injury; pregnancy or breastfeeding; an anticipated life expectancy of <3 months; or participation in another interventional study. Stable comorbidities were not exclusion criteria unless they precluded safe application of RIC or completion of the study procedures; baseline comorbidities are summarized in Table 1.

### 2.2. Procedures

Transthoracic echocardiography and hemodynamic measurements during RHC were obtained immediately before and after the RIC intervention. Echocardiography was performed according to a standardized protocol to assess RV structure and function. Detailed echocardiographic measurements, including tricuspid annular plane systolic excursion, tricuspid annular systolic velocity, tricuspid regurgitation indices, left ventricular eccentricity index, and hepatic venous flow parameters, are provided in the Supplementary Methods.

RHC was performed via the right internal jugular vein using a Swan–Ganz catheter without procedural sedation. Hemodynamic measurements included right atrial pressure and pulmonary arterial pressures, pulmonary capillary wedge pressure, and cardiac output. Cardiac output was determined by thermodilution as the mean of three consecutive measurements. Pulmonary and systemic vascular resistances were calculated using standard formulas. Detailed hemodynamic procedures and calculations of oxygen transport indices, including arterial and mixed venous oxygen content, oxygen delivery, oxygen consumption, oxygen extraction ratio, and intrapulmonary shunt fraction, are provided in the Supplementary Methods.

**Figure 1:**
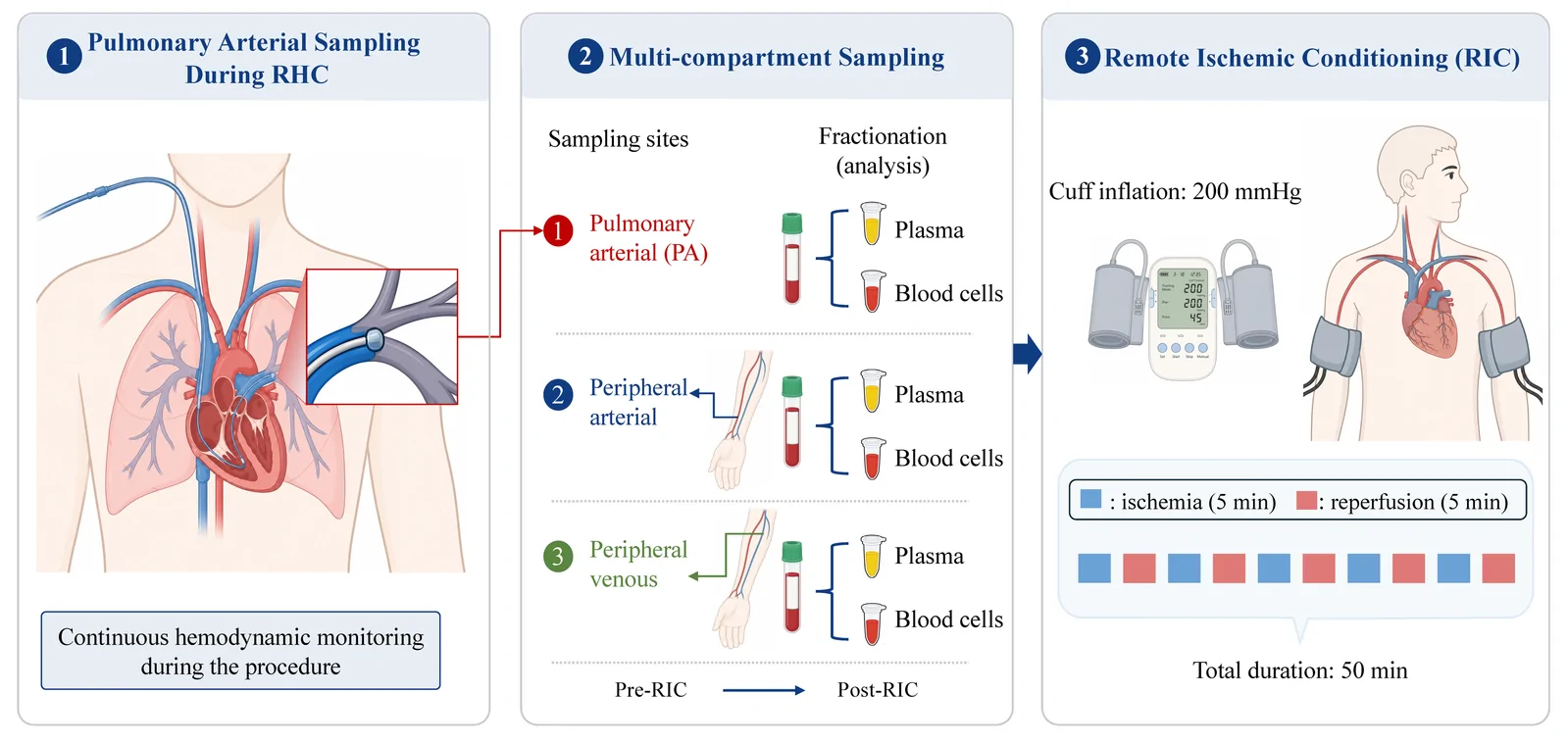
Study design and sampling protocol. Pulmonary arterial blood was collected immediately before and after RIC, whereas peripheral arterial and venous blood was collected before and 60 min after RIC. RIC comprised five cycles of 5 min ischemia and 5 min reperfusion. Illustrative elements in this figure were generated using ChatGPT Images (GPT-Image-1.5; OpenAI, San Francisco, CA, USA) and subsequently arranged, labeled, and edited by the authors using Microsoft PowerPoint for Mac, version 16.108.3 (Microsoft Corporation, Redmond, WA, USA). Abbreviations: RHC, right heart catheterization; RIC, remote ischemic conditioning.

For compartment-specific assessment of redox biomarkers, blood samples were obtained from the pulmonary artery through the Swan–Ganz catheter and from a peripheral artery and vein. PA blood was collected immediately before and immediately after RIC. Peripheral arterial and venous blood was collected before RIC and 60 min after completion of RIC because repeated upper-limb ischemia precluded reliable immediate post-RIC peripheral sampling (Figure 1). PA and peripheral arterial samples were used for blood-gas analysis, whereas samples from all three vascular compartments were analyzed for redox-related biomarkers.

### 2.3. Remote Ischemic Conditioning

Following placement of the Swan–Ganz catheter, cuff straps were applied to both upper limbs and inflated to a pressure of 200 mmHg. The RIC protocol consisted of 5 cycles of ischemia–reperfusion, each cycle comprising 5 min of cuff inflation followed by 5 min of deflation, for a total duration of 50 min (Figure 1). The procedure was performed using an automated cuff inflation device (Patent No. CN200820123637.X, China). A similar method has been described by other authors [26]. Heart rate and systemic arterial pressure were continuously monitored throughout the procedure. The intervention was immediately discontinued if the participant reported intolerable pain or any significant discomfort.

### 2.4. Sample Processing and Redox Biomarker Analysis

Blood samples for redox biomarker analysis were collected into heparinized tubes and processed within 1 h of collection. Samples were centrifuged at 1200× *g* for 10 min at 4 °C, after which the plasma supernatant and the remaining blood-cell fraction were separated and stored at −80 °C until batch analysis. The blood-cell fraction was analyzed as a mixed cellular fraction without further separation into erythrocyte, leukocyte, or platelet subpopulations.

Redox-related metabolites in plasma and blood-cell fractions were measured using a targeted ultra-performance liquid chromatography–mass spectrometry redox metabolomics method adapted from Cai et al. [27]. An isotope-labeled glutathione internal standard (GSH–^13^_2_^15^_12_^15^N_1_) was used for normalization. Because the available sample volume varied among samples, analyte-to-internal-standard peak-area ratios were further normalized to the actual sample volume used for extraction. Accordingly, the results are reported as volume-normalized relative response units rather than absolute metabolite concentrations. In this manuscript, “GSH response” refers to the volume-normalized analyte-to-internal-standard signal used to represent GSH abundance and does not denote a causal treatment response. Detailed sample extraction procedures and analytical methods are provided in the Supplementary Methods.

### 2.5. Outcome Assessments

The prespecified primary biomarker family comprised four PA glutathione-related measures: GSH response and the GSSG/GSH ratio in the blood-cell and plasma fractions. Changes were assessed from immediately before to immediately after RIC.

Pulmonary arterial lactate concentration was the prespecified secondary endpoint. Exploratory endpoints included glutathione-related measures in peripheral arterial and venous blood, additional redox-related biomarkers across vascular compartments, right ventricular function assessed by echocardiography, blood-gas parameters, oxygen transport indices, and hemodynamic variables.

Short-term tolerability was assessed by monitoring adverse events during and after RIC, including inability to tolerate the intervention, acute hemodynamic instability, clinically evident tissue or neurovascular injury, as well as changes in N-terminal pro–B-type natriuretic peptide (NT-proBNP) from baseline to 12–24 h after RIC.

### 2.6. Statistical Analysis

Statistical analyses were performed using R software (version 4.5.1; R Foundation for Statistical Computing, Vienna, Austria) and GraphPad Prism version 10.5.0 (GraphPad Software, Boston, MA, USA). Given the exploratory pilot design, no formal sample size calculation was performed; the sample size (*n* = 15) was determined by recruitment and procedural feasibility. Continuous variables are presented as mean ± standard deviation or median (interquartile range), as appropriate, and categorical variables as number (percentage).

For paired analyses, differences were defined as post-RIC minus pre-RIC values, and the distribution of the paired differences was assessed using the Shapiro–Wilk test. Outcomes with approximately normally distributed paired differences were compared using paired t-tests and are reported as mean paired differences with 95% confidence intervals (CIs). Outcomes with non-normally distributed paired differences were compared using Wilcoxon signed-rank tests and are reported as median paired differences with percentile-bootstrap 95% CIs based on 2000 resamples of the paired differences.

The four prespecified primary PA glutathione-related measures—GSH response and the GSSG/GSH ratio in PA blood-cell and plasma fractions—constituted a single multiplicity family. Their nominal *p* values were adjusted using Holm’s sequential procedure to control the family-wise error rate. A primary component was considered statistically significant when its Holm-adjusted *p* value was <0.05.

Pulmonary arterial lactate was the sole prespecified secondary endpoint and was evaluated without further multiplicity adjustment. All other outcomes, including peripheral glutathione-related measures, additional redox-related metabolites, echocardiographic variables, blood gas parameters, oxygen transport indices, and hemodynamic variables, were considered exploratory. *p* values for these exploratory analyses were not adjusted for multiple comparisons and are reported as nominal; these findings should therefore be interpreted as hypothesis-generating.

All statistical tests were two-sided. Missing values were not imputed, and each analysis was based on participants with available paired pre- and post-RIC measurements. Given the pilot design, interpretation emphasized paired effect estimates and 95% CIs. The detailed statistical analysis strategy, including Holm adjustment across the primary biomarker family, was finalized before database lock and outcome analysis.

## 3. Results

### 3.1. Participant Characteristics

Seventeen patients with PH were screened, of whom 15 were enrolled and completed the study protocol. The cohort included 10 patients with idiopathic pulmonary arterial hypertension, three with connective tissue disease-associated pulmonary arterial hypertension, and two with CTEPH. The mean age was 44 ± 13 years, 12 participants (80%) were female, the mean body mass index was 23 ± 3 kg/m^2^ and the mean six-minute walk distance was 409 ± 140 m (Table 1).

### 3.2. Prespecified Primary Pulmonary Arterial Glutathione Outcomes

Following RIC, PA blood-cell GSH response increased (mean paired difference, 118; 95% CI, 83 to 152; Holm-adjusted *p* < 0.001), whereas the PA blood-cell GSSG/GSH ratio decreased (mean paired difference, −0.64; 95% CI, −0.81 to −0.46; Holm-adjusted *p* < 0.001).

**Figure 2:**
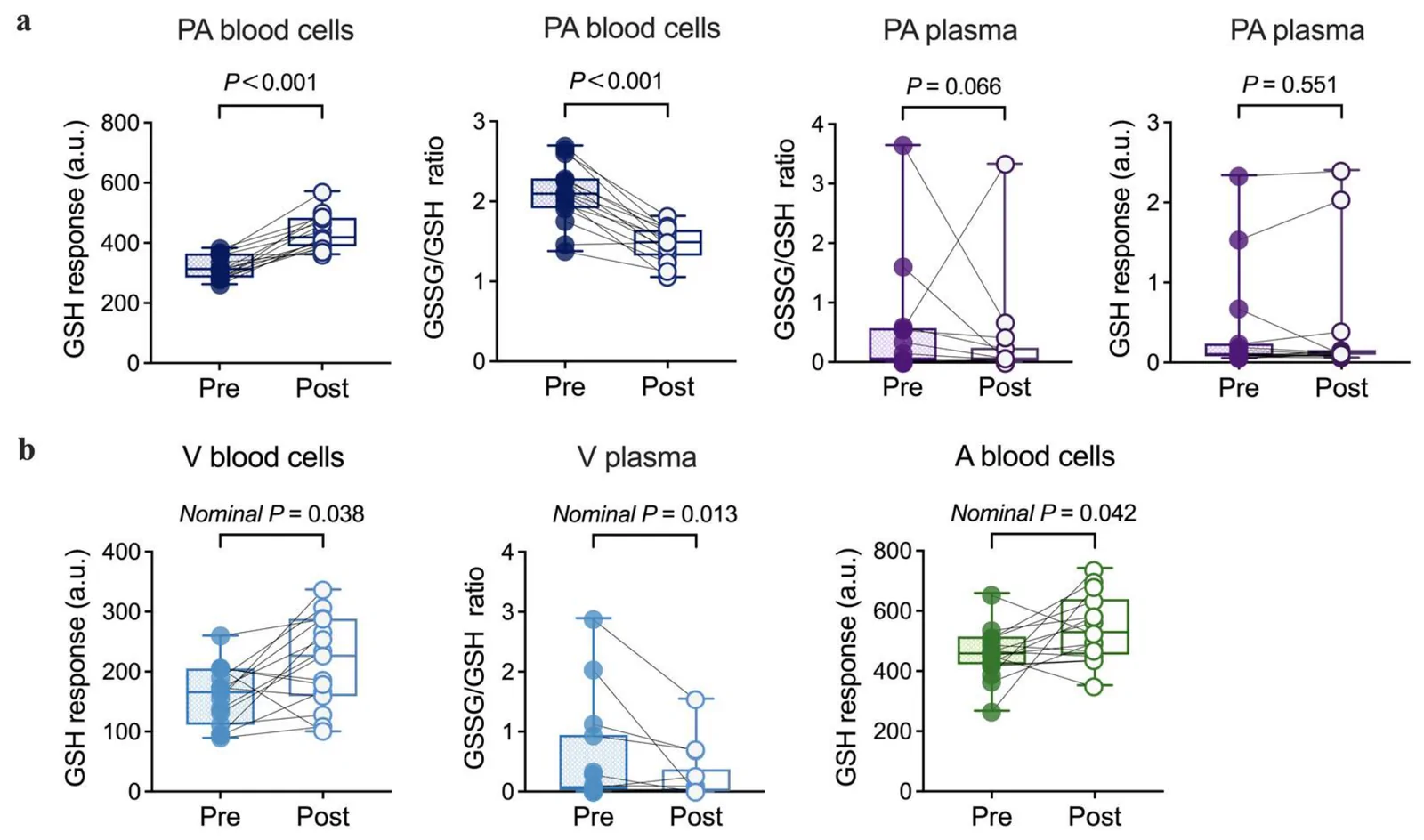
Glutathione redox responses after remote ischemic conditioning. (**a**) Changes in the four prespecified PA glutathione component markers before and immediately after RIC, including GSH response and the GSSG/GSH ratio in PA blood cells and PA plasma. Displayed *p* values are Holm-adjusted for the primary endpoint family. (**b**) Exploratory peripheral blood glutathione markers with nominal changes (nominal *p* < 0.05) before and 60 min after RIC, including venous blood-cell GSH response, arterial blood-cell GSH response, and venous plasma GSSG/GSH ratio. Lines indicate paired individual changes; box plots show the median and interquartile range. All *p* values in panel (**b**) are nominal and should be interpreted as hypothesis-generating. Abbreviations: GSH, reduced glutathione; GSSG, oxidized glutathione; PA, pulmonary arterial; RIC, remote ischemic conditioning.

In the PA plasma fraction, the GSSG/GSH ratio showed a nominal decrease (median paired difference, −0.013; 95% CI, −0.124 to −0.002; nominal *p* = 0.030), but this change did not remain statistically significant after Holm adjustment (adjusted *p* = 0.066). PA plasma GSH response did not show evidence of change (median paired difference, 0.002; 95% CI, −0.066 to 0.024; nominal *p* = 0.551; Holm-adjusted *p* = 0.551). These findings are shown in Figure 2a and summarized in Supplementary Table S1.

### 3.3. Secondary Endpoint: Pulmonary Arterial Lactate

**Figure 3:**
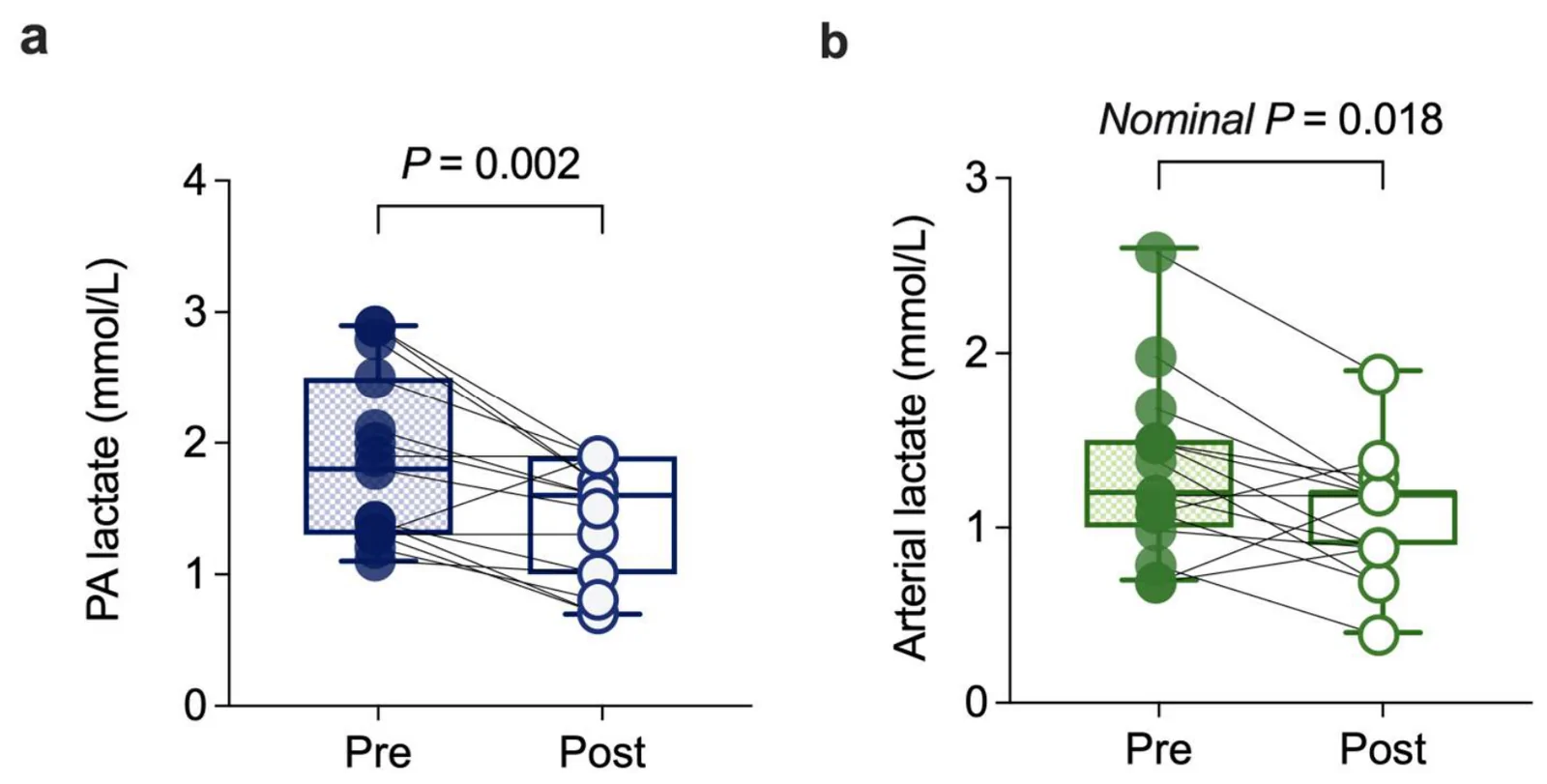
Pulmonary arterial and peripheral arterial lactate responses after remote ischemic conditioning. (**a**) Changes in PA lactate before and immediately after RIC (0 min); PA lactate was the sole prespecified secondary endpoint. (**b**) Changes in peripheral arterial lactate before RIC and at 60 min after RIC completion; the *p* value in panel (**b**) is nominal and hypothesis-generating. Lines indicate paired individual changes; box plots show the median and interquartile range. Abbreviations: PA, pulmonary arterial; RIC, remote ischemic conditioning.

PA lactate, the sole prespecified secondary endpoint, decreased after RIC (paired difference, −0.45 mmol/L; 95% CI, −0.71 to −0.20; *p* = 0.002) (Figure 3a and Table 2).

### 3.4. Exploratory Peripheral Redox Responses

At 60 min after RIC, peripheral blood-cell GSH response showed nominal increases in both venous samples (mean paired difference, 52.0; 95% CI, 3.2 to 101.1; nominal *p* = 0.038) and arterial samples (mean paired difference, 87.1; 95% CI, 3.8 to 170.4; nominal *p* = 0.042). The peripheral venous plasma GSSG/GSH ratio also showed a nominal decrease (median paired difference, −0.05; 95% CI, −0.338 to −0.002; nominal *p* = 0.013).

Other peripheral glutathione-related measures did not yield nominal *p* values < 0.05, including the blood-cell GSSG/GSH ratio in venous (paired difference, −0.22; 95% CI, −0.49 to 0.06; *p* = 0.113) and arterial samples (paired difference, −0.06; 95% CI, −0.41 to 0.28; *p* = 0.702), arterial plasma GSH response (*p* = 0.721), arterial plasma GSSG/GSH ratio (*p* = 0.624), and venous plasma GSH response (*p* = 0.629). These exploratory findings are shown in Figure 2b and Supplementary Table S2.

Because PA samples were obtained immediately after RIC whereas peripheral arterial and venous samples were obtained 60 min after RIC completion, no formal comparison of effect magnitudes across vascular compartments or post-intervention time points was performed.

### 3.5. Exploratory Physiological Responses

Among exploratory physiological outcomes, tricuspid annular plane systolic excursion showed a nominal increase (paired difference, 0.3 mm; 95% CI, 0 to 0.5; nominal *p* = 0.030). Other echocardiographic measures did not show nominal evidence of change.

Among blood-gas variables, PA pH showed a nominal increase (*p* = 0.035), whereas mixed venous carbon dioxide partial pressure and mixed venous oxygen partial pressure showed nominal decreases (*p* = 0.041 and *p* = 0.013, respectively). Peripheral arterial lactate also showed a nominal decrease (*p* = 0.018). No measured hemodynamic or oxygen-transport variable yielded a nominal *p* value < 0.05. These findings are summarized in Table 2 and Supplementary Table S3.

### 3.6. Exploratory Redox-Related Biomarkers

Beyond the glutathione system, nominal increases were observed in PA blood-cell NADP^+^ (*p* < 0.001), the PA blood-cell NAD^++^/NADPH ratio (*p* = 0.003), PA blood-cell NADH (*p* = 0.025), peripheral venous plasma arginine (*p* = 0.011), and peripheral venous plasma cysteine (*p* = 0.021). No consistent pattern was observed among the remaining NAD/NADP-related metabolites. Detailed exploratory redox-related biomarker results are provided in Supplementary Table S2. These unadjusted exploratory findings should be interpreted as hypothesis-generating.

### 3.7. Short-Term Tolerability

All participants completed and tolerated the RIC intervention without acute hemodynamic instability or clinically evident tissue or neurovascular injury during the observation period. NT-proBNP did not show a detectable paired change from baseline to 12–24 h after RIC (paired difference, −3.7 pg/mL; 95% CI, −151 to 33; *p* = 0.454).

## 4. Discussion

In this prospective, open-label, single-arm biomarker pilot study, a single 50 min session of RIC was followed by acute changes in PA glutathione-related biomarkers in patients with PH. Among the four prespecified primary biomarker components, PA blood-cell GSH response increased and the GSSG/GSH ratio decreased after Holm adjustment, whereas the corresponding plasma measures did not remain statistically significant after multiplicity correction. PA lactate, the prespecified secondary endpoint, also decreased. In contrast, no consistent changes were observed in hemodynamic or oxygen-transport variables. These findings suggest that acute redox and metabolic changes in PA blood may be detectable in the absence of concurrent macrohemodynamic responses. However, given the single-arm design and small sample size, the observed changes cannot be attributed causally to RIC and should be regarded as preliminary and hypothesis-generating.

A notable feature of this study was the direct sampling of PA blood during right heart catheterization. Previous human studies of ischemic conditioning and pulmonary vascular physiology have primarily examined healthy volunteers exposed to acute hypoxia and focused on pulmonary artery pressure or oxygenation [23,24,25]. In contrast, the present study evaluated patients with established PH and directly characterized paired glutathione-related changes in pulmonary arterial blood. This direct pulmonary arterial redox assessment extends the existing human literature from physiological responses in healthy volunteers to compartment-specific biochemical observations in PH. Most human studies investigating oxidative stress in PH rely on peripheral blood, whereas direct pulmonary arterial redox measurements remain uncommon [14,15]. Direct PA sampling therefore provided an opportunity to assess glutathione-related changes in a vascular compartment that is rarely examined in interventional studies. Importantly, PA blood biomarkers cannot be considered direct surrogates of pulmonary vascular tissue redox biology. Moreover, PA samples were obtained immediately after RIC, whereas peripheral arterial and venous samples were obtained 60 min after RIC completion. Therefore, differences between PA and peripheral responses cannot be separated from differences in sampling time, and no inference regarding the relative magnitude or duration of compartment-specific responses can be made from the present data.

The glutathione response was more evident in the blood-cell fraction than in plasma. This finding is biologically plausible because glutathione is predominantly intracellular, with substantially higher concentrations in circulating blood cells, particularly erythrocytes, than in plasma [28]. The cellular fraction may therefore provide a larger glutathione pool in which acute changes are more readily detectable, whereas plasma glutathione measurements are more susceptible to cellular leakage and preanalytical handling [28,29]. Nevertheless, the blood-cell fraction in this study represented a mixed cellular pellet containing erythrocytes, leukocytes, and platelets. The specific cellular population responsible for the observed signal therefore cannot be determined. In addition, plasma glutathione measurements may be particularly susceptible to oxidation and contamination from cellular components because of their relatively low circulating abundance [28,30]. These analytical and biological differences should be considered when interpreting the stronger response in blood-cell than in plasma.

One potential pathway linking RIC to glutathione-related changes is Nrf2 signaling. This pathway may be particularly relevant in PH, in which oxidative stress and disturbances in glutathione-related metabolism have been implicated in pulmonary vascular and RV remodeling [6,13]. Within the glutathione redox cycle, GSH is oxidized to GSSG during peroxide reduction and can subsequently be regenerated through NADPH-dependent glutathione reductase activity [31]. Experimental studies have reported activation of the Nrf2/heme oxygenase-1 pathway after ischemic conditioning, and Nrf2 regulates multiple genes involved in glutathione synthesis, recycling, and cellular antioxidant defense [18,32]. In addition, human data indicate that remote ischemic preconditioning can modify erythrocyte GSH responses during experimental ischemia–reperfusion, providing clinical evidence that the glutathione system may participate in the systemic biological response to ischemic conditioning [33]. Importantly, the concurrent increase in PA blood-cell GSH response and decrease in the GSSG/GSH ratio observed in our study are consistent with a shift toward a more reduced glutathione state within the mixed blood-cell fraction following RIC. The exploratory increase in peripheral venous plasma cysteine at 60 min may also be compatible with altered substrate availability, although differences in compartment and sampling time preclude linking this change directly to the immediate PA glutathione findings. However, Nrf2 activation, glutathione-synthetic enzymes, and metabolic flux were not directly measured. Accordingly, the present findings do not demonstrate activation of the Nrf2 pathway or increased glutathione synthesis. Rather, they identify an acute change in glutathione-related indices that is compatible with, but does not establish, altered cellular redox regulation after RIC.

The decrease in PA lactate provides an additional metabolic observation. Lactate reflects not only tissue perfusion but also cellular glycolytic activity, redox balance, and mitochondrial metabolism [34,35,36]. In the present study, PA lactate decreased without detectable changes in cardiac output, pulmonary vascular resistance, oxygen delivery, or oxygen extraction, making an acute improvement in global oxygen transport an unlikely explanation for the observed change. Because lactate metabolism is closely linked to cytosolic NADH/NAD^+^ balance [37], the concurrent changes in PA lactate and blood-cell glutathione indices may indicate an early redox–metabolic response. However, mitochondrial function and metabolic flux were not directly assessed, and the mechanistic relationship between these observations remains uncertain. Similarly, the nominal change in tricuspid annular plane systolic excursion and other exploratory physiological findings should be interpreted cautiously because they were not adjusted for multiple comparisons and may reflect measurement variability or chance. The absence of consistent hemodynamic changes further indicates that a single RIC session did not produce a measurable immediate change in integrated cardiopulmonary function under the conditions studied.

The intervention was completed by all participants without clinically evident intolerance, acute hemodynamic instability, tissue or neurovascular injury, or an increase in N-terminal pro–B-type natriuretic peptide during short-term observation. These findings support the feasibility and short-term tolerability of the protocol in this small, carefully monitored cohort but cannot establish safety or exclude uncommon adverse events.

Several additional limitations should be acknowledged. First, the study was conducted at a single center, included only 15 participants, and lacked a sham-control group and blinding; therefore, temporal effects related to the procedure, repeated sampling, or regression to the mean cannot be excluded. Second, only the acute response following a single RIC session was evaluated, and the persistence or reproducibility of the observed biomarker changes is unknown. Third, redox metabolite measurements were reported as volume-normalized relative responses rather than absolute concentrations, limiting direct quantitative comparison with studies using absolute metabolite concentrations, and the mixed blood-cell fraction did not allow identification of the cellular source of the glutathione signal. Fourth, the study included patients with heterogeneous PH etiologies, which further limits generalizability. In addition, stable comorbidities may have contributed to inter-individual variability in redox biomarkers; however, the small sample size precluded meaningful adjustment for these potential confounders. Finally, although ethical approval was obtained before recruitment, the study was retrospectively registered in the Chinese Clinical Trial Registry after recruitment had commenced but before enrollment of the final participant, database lock, and outcome analysis. The primary and secondary outcomes had been specified in the ethics-approved study protocol before enrollment of the first participant and were not modified thereafter.

These preliminary findings warrant confirmation in randomized sham-controlled studies designed to determine whether the observed glutathione-related changes are reproducibly attributable to RIC. Future studies should incorporate prospectively registered protocols, repeated RIC sessions, synchronized pulmonary and peripheral blood sampling, and more detailed characterization of cellular redox pathways, including cell-specific analyses and, where feasible, absolute metabolite quantification or metabolic-flux measurements. Although these acute biochemical changes were not accompanied by consistent hemodynamic or oxygen-transport changes, they provide a biologically plausible redox–metabolic signal that supports further randomized sham-controlled and longer-term intervention studies to determine their reproducibility and whether they translate into sustained physiological or clinical benefits.

## 5. Conclusions

In this single-arm biomarker pilot study, a single RIC session was well tolerated and was followed by acute changes in pulmonary arterial blood-cell glutathione-related indices and a reduction in pulmonary arterial lactate in patients with PH. These preliminary findings suggest an acute redox–metabolic signal following RIC, but the absence of a sham control and the small sample size preclude causal inference. Further randomized sham-controlled and longer-term studies are warranted to determine the reproducibility and potential clinical relevance of these redox–metabolic changes.

## Figures and Tables

**Table 1:** Baseline Characteristics.

Characteristic	Patients (*N* = 15)
Age (y)	44 ± 13
Female, *n* (%)	12 (80)
Height (m)	1.62 ± 0.06
Weight (kg)	61 ± 10
BMI (kg/m^2^)	23 ± 3
6MWD (m)	409 ± 140
Hemoglobin (g/L)	135 ± 27
NT-proBNP (pg/mL)	552 (217–1158)
PH classification, *n* (%)	
CTEPH	2 (13)
CTD-PAH	3 (20)
IPAH	10 (67)
Medications, *n* (%)	
Endothelin Receptor Antagonist	13 (87)
Soluble Guanylate Cyclase Stimulator	8 (53)
Selective Prostacyclin IP Receptor Agonist	10 (67)
Factor Xa Inhibitor/Direct Oral Anticoagulant	4 (27)
Phosphodiesterase Type 5 Inhibitor	1 (7)
Prostacyclin Analogs/Prostanoids	2 (13)
WHO-FC, *n* (%)	
II	7 (47)
III	7 (47)
IV	1 (7)
Medical history, *n* (%)	
Diabetes	1 (7)
Hypertension	3 (20)
Hyperlipidemia	2 (13)

Abbreviations: BMI, body mass index; 6MWD, 6 min walk distance; WHO-FC, World Health Organization functional class; NT-proBNP, N-terminal pro–B-type natriuretic peptide; PH, pulmonary hypertension; PAH, pulmonary arterial hypertension; CTD-PAH, connective tissue disease-associated pulmonary arterial hypertension; IPAH, idiopathic pulmonary arterial hypertension; CTEPH, chronic thromboembolic pulmonary hypertension.

**Table 2:** Echocardiographic and Blood-Gas Variables Before and After Remote Ischemic Conditioning. Continuous variables are presented as mean ± standard deviation or median (interquartile range), as appropriate. Paired differences were calculated as post-RIC minus pre-RIC values. Bold type indicates the prespecified secondary endpoint. All other comparisons were exploratory, and their *p* values are nominal and were not adjusted for multiple comparisons.

Variables	Pre-RIC (*N* = 15)	Post-RIC (*N* = 15)	Mean/Median Paired Difference (95% CI)	*p* Value
**Echocardiography**				
EI	1.43 ± 0.32	1.45 ± 0.29	0.02 (−0.04 to 0.08)	0.516
TRV (cm/s)	423 ± 80	450 ± 96	26.2 (−2.6 to 55.0)	0.071
TRPG (mmHg)	72 (67–86)	84 (63–99)	5.0 (−5.0 to 20.0)	0.105
TAPSE (mm)	16.1 (12.5–18.2)	16.6 (12.9–18.3)	0.3 (0.0 to 0.5)	0.030
S’ (cm/s)	10.0 ± 1.6	10.2 ± 1.4	0.2 (−0.2 to 0.6)	0.315
Vmax (cm/s)	41 ± 9	38 ± 8	−3.0 (−9.0 to 2.9)	0.295
VTI (cm)	5.2 ± 1.8	5.1 ± 2.1	−0.1 (−1.0 to 0.8)	0.815
**Blood Gas**				
A-Lac (mmol/L)	1.3 ± 0.5	1.1 ± 0.4	−0.27 (−0.48 to −0.05)	0.018
**PA-Lac (mmol/L)**	**1.9 ± 0.7**	**1.4 ± 0.5**	**−0.45 (−0.71 to −0.20)**	**0.002**
A-pH	7.45 ± 0.03	7.44 ± 0.04	−0.01 (−0.03 to 0.01)	0.380
PA-pH	7.36 ± 0.04	7.38 ± 0.03	0.03 (0.01 to 0.05)	0.035
PmvCO_2_ (mmHg)	46.6 ± 5.2	43.9 ± 3.2	−3.4 (−5.7 to −1.0)	0.041
PmvO_2_ (mmHg)	37.1 ± 3.2	35.5 ± 3.4	−1.6 (−2.9 to −0.3)	0.013
PaO_2_ (mmHg)	69.2 ± 10.3	66.0 ± 9.1	−3.2 (−8.1 to 1.7)	0.186
PaCO_2_ (mmHg)	34.5 ± 3.1	35.7 ± 3.1	1.2 (−0.2 to 2.6)	0.084
SvO_2_ (%)	63.8 ± 7.6	62.3 ± 8.5	−1.2 (−3.2 to 0.9)	0.239
SaO_2_ (%)	94.2 (93.3–95.3)	94.0 (92.3–94.5)	−0.2 (−0.8 to 0.9)	0.594

Abbreviations: EI, eccentricity index; TRV, tricuspid regurgitation velocity; TRPG, tricuspid regurgitation pressure gradient; TAPSE, tricuspid annular plane systolic excursion; S′, systolic tricuspid annular velocity; VTI, velocity–time integral; A-Lac, arterial lactate; PA-Lac, pulmonary arterial lactate; PaO_2_, arterial oxygen partial pressure; PaCO_2_, arterial carbon dioxide partial pressure; PmvO_2_, mixed venous oxygen partial pressure measured in pulmonary arterial blood; PmvCO_2_, mixed venous carbon dioxide partial pressure measured in pulmonary arterial blood; SaO_2_, arterial oxygen saturation; SvO_2_, mixed venous oxygen saturation measured in pulmonary arterial blood.

## Data Availability

The data that support the findings of this study are available upon request from the corresponding author.

## Supplementary Materials

The following supporting information can be downloaded at https://www.mdpi.com/article/10.3390/antiox15091203/s1; Table S1: Prespecified Pulmonary Arterial Glutathione Biomarker Outcomes Before and After Remote Ischemic Conditioning; Table S2: Exploratory Redox-Related Biomarker Changes Before and After Remote Ischemic Conditioning; Table S3: Hemodynamic and Oxygen-Transport Variables Before and After Remote Ischemic Conditioning.

## Abbreviations

The following abbreviations are used in this manuscript:

CIs	Confidence intervals
CO	Cardiac output
CTEPH	Chronic thromboembolic pulmonary hypertension
GSH	Reduced glutathione
GSSG	Oxidized glutathione
mPAP	Mean pulmonary arterial pressure
NAD^+^	Nicotinamide adenine dinucleotide
NADH	Reduced nicotinamide adenine dinucleotide
NADP^+^	Nicotinamide adenine dinucleotide phosphate
Nrf2	Nuclear factor erythroid 2–related factor 2
NADPH	Reduced nicotinamide adenine dinucleotide phosphate
NT-proBNP	N-terminal pro-B-type natriuretic peptide
PA	Pulmonary arterial
PCWP	Pulmonary capillary wedge pressure
PH	Pulmonary hypertension
PVR	Pulmonary vascular resistance
RV	Right ventricle
RHC	Right heart catheterization
RIC	Remote ischemic conditioning
SVR	Systemic vascular resistance
TAPSE	Tricuspid annular plane systolic excursion
