# Supplementary material for: Glutathione Redox Changes in Pulmonary Arterial Blood After Remote Ischemic Conditioning in Pulmonary Hypertension: A Single-Arm Pilot Study
Source: Antioxidants (Basel). 2026 Sep 19;15(9):1203. doi: 10.3390/antiox15091203 (PMC13603679; doi:10.3390/antiox15091203)
Supplement: Supplementary file 1 [file antioxidants-15-01203-s001.zip › antioxidants-4540006-supplementary.pdf]

Echocardiographic Assessment

Transthoracic echocardiography was performed immediately before and after remote ischemic conditioning using a standardized protocol and a Philips EPIQ 7C ultrasound system (Philips Healthcare, USA) equipped with an X5-1 transducer (1 to 5 MHz). Participants were examined in the left lateral decubitus position by echocardiographers with at least 3 years of experience.

Right ventricular longitudinal systolic function was assessed using tricuspid annular plane systolic excursion (TAPSE) and peak systolic velocity of the lateral tricuspid annulus (S′). TAPSE was measured using M-mode imaging as the systolic displacement of the lateral tricuspid annulus, and S′ was measured using tissue Doppler imaging. Tricuspid regurgitation velocity was measured using continuous-wave Doppler, and the tricuspid regurgitation pressure gradient was calculated using the simplified Bernoulli equation.

The left ventricular eccentricity index was measured from the parasternal short-axis view at end systole and end diastole. Hepatic venous flow was assessed using pulsed-wave Doppler, including the maximal velocity and velocity–time integral of hepatic vein systolic flow reversal. Measurements were obtained over three cardiac cycles and analyzed by investigators who were not blinded to the sampling time point. The same acquisition and measurement methods were used before and after RIC.

Right Heart Catheterization and Hemodynamic Assessment

Right heart catheterization was performed at the bedside through the right internal

jugular vein using a Swan–Ganz catheter. No procedural sedation, digital subtraction angiography, or radiographic contrast was used. The catheter position was confirmed using pressure-waveform changes.

Heart rate, systemic arterial pressure, right atrial pressure, right ventricular systolic and end-diastolic pressures, systolic, diastolic, and mean pulmonary arterial pressures, and pulmonary capillary wedge pressure were recorded. Pressure measurements were obtained at end-expiration and averaged over three cardiac cycles.

Cardiac output was measured using the thermodilution method. Three consecutive measurements differing by no more than 10% were averaged. Pulmonary vascular resistance was calculated as:

$$PVR = 80 \times (mPAP - PCWP) / CO$$

Systemic vascular resistance was calculated as:

$$SVR = 80 \times (MAP - RAP) / CO$$

where PVR is pulmonary vascular resistance, mPAP is mean pulmonary arterial pressure, PCWP is pulmonary capillary wedge pressure, CO is cardiac output, SVR is systemic vascular resistance, MAP is mean systemic arterial pressure, and RAP is right atrial pressure. Resistance values were expressed in $dyn \cdot s \cdot cm^{-5}$.

Blood-Gas Analysis and Oxygen-Transport Calculations

Peripheral arterial and pulmonary arterial mixed-venous blood gases were measured using an ABL90 FLEX blood-gas analyzer (Radiometer Medical ApS, Denmark). To

avoid confusion with peripheral venous samples, pulmonary arterial mixed-venous oxygen and carbon dioxide tensions are denoted as $PmvO_2$ and $PmvCO_2$, respectively.

Oxygen-transport variables included the alveolar–arterial oxygen tension difference ($AaDO_2$), arterial oxygen content (CaO_2), mixed-venous oxygen content ($CmvO_2$), arteriovenous oxygen-content difference ($Ca{-}mvO_2$), oxygen delivery (DO_2), oxygen consumption (VO_2), oxygen-extraction ratio (O_2ER), and estimated intrapulmonary shunt fraction (Qs/Qt).

Alveolar oxygen partial pressure was calculated using the alveolar gas equation:

$$PAO_2 = FiO_2 \times (PB - PH_2O) - PaCO_2/R$$

where FiO_2 is the inspired oxygen fraction, PB is barometric pressure, PH_2O is water-vapor pressure, $PaCO_2$ is arterial carbon dioxide tension, and R is the respiratory exchange ratio. For measurements performed on room air, FiO_2 was 0.21, PB was approximately 760 mmHg, PH_2O was 47 mmHg at 37 °C, and R was assumed to be 0.8.

The following equations were used:

$$AaDO_2 = PAO_2 - PaO_2$$

$$CaO_2 = (1.34 \times Hb \times SaO_2) + (0.003 \times PaO_2)$$

$$CmvO_2 = (1.34 \times Hb \times SmvO_2) + (0.003 \times PmvO_2)$$

$$Ca{-}mvO_2 = CaO_2 - CmvO_2$$

$$DO_2 = CO \times CaO_2 \times 10$$

$VO_2 = CO \times (CaO_2 - CmvO_2) \times 10$

$O_2ER = (CaO_2 - CmvO_2) / CaO_2$

$CcO_2 = (1.34 \times Hb) + (0.003 \times PAO_2)$

$Qs/Qt = (CcO_2 - CaO_2) / (CcO_2 - CmvO_2) \times 100\%$

In these equations, PaO_2 and $PmvO_2$ are arterial and pulmonary arterial mixed-venous oxygen tensions in mmHg; SaO_2 and $SmvO_2$ are the corresponding oxygen saturations expressed as fractions; Hb is hemoglobin concentration in g/dL; and CO is cardiac output in L/min. The constants 1.34 and 0.003 represent the Hüfner constant and the solubility of oxygen in plasma, respectively. The factor of 10 converts cardiac output from L/min to dL/min, allowing DO_2 and VO_2 to be expressed in mL/min.

Detailed Sample Extraction and UPLC–MS Analysis

Blood samples were collected into heparinized tubes and processed as described in the main text. After separation of plasma and the mixed blood-cell fraction, a measured aliquot of each sample was used for metabolite extraction. Because the available sample volume varied among samples, the exact aliquot volume used for each extraction was recorded for subsequent normalization.

Redox-related metabolites were analyzed using a targeted redox metabolomics method adapted from Cai et al. Each sample aliquot was mixed with 400 µL of ice-cold 80% methanol containing the isotope-labeled internal standard GSH-$^{13}C_2{}^{15}N_1$ (1 µL, 1 mg/mL), followed by addition of 1 mL methyl tert-butyl ether. After vortex

mixing for 10 min, 150 μL of triple-distilled water was added to facilitate phase separation. Samples were centrifuged at 12,000 × g for 10 min at 4 °C. The lower aqueous phase was collected and filtered through a 0.22-μm nylon membrane filter, and 5 μL of filtrate was injected into the UPLC–MS system under chromatographic and mass-spectrometric conditions adapted from the previously described method[26].

Relative metabolite abundance was determined from the analyte-to-internal-standard peak-area ratio and was further normalized to the actual sample volume used for extraction:

$$R_V = \frac{A_{analyte}}{A_{IS} \times V_{sample}}$$

where R_V represents the volume-normalized relative response, $A_{analyte}$ is the analyte peak area, A_{IS} is the internal-standard peak area, and V_{sample} is the actual sample volume used for extraction. Results are therefore reported as volume-normalized relative response units rather than absolute metabolite concentrations.

Table S1. Prespecified Pulmonary Arterial Glutathione Biomarker Outcomes Before and After Remote Ischemic Conditioning.

Biomarker	Compartment	Pre-RIC (N=15)	Post-RIC (N=15)	Mean/Median Difference (95% CI)	Nominal *P*	Holm-adjusted *P*
GSH	PA blood cells	320 ± 40	438 ± 59	117.5 (83.1 to 152.0)	<0.001	<0.001
GSSG/GSH	PA blood cells	2.1 ± 0.4	1.5 ± 0.2	−0.64 (−0.81 to −0.46)	<0.001	<0.001
GSH	PA plasma	0.10 (0.08, 0.22)	0.11 (0.10, 0.14)	0.002 (−0.066 to 0.024)	0.551	0.551
GSSG/GSH	PA plasma	0.059 (0.023, 0.564)	0.057 (0.023, 0.162)	−0.013 (−0.124 to −0.002)	0.030	0.066

Redox biomarkers are reported as analyte-to-internal-standard peak-area ratios normalized to the actual sample volume used for extraction and are expressed as volume-normalized relative response units rather than absolute concentrations.

Abbreviations: GSH, reduced glutathione; GSSG, oxidized glutathione; PA, pulmonary arterial; RIC, remote ischemic conditioning.

Table S2. Exploratory Redox-Related Biomarker Changes Before and After Remote Ischemic Conditioning.

Biomarker	Compartment	Pre-RIC (N=15)	Post-RIC (N=15)	Mean/Median Difference (95% CI)	Nominal *P*
GSH	V blood cells	160 ± 50	212 ± 82	52.0 (3.2 to 101.1)	0.038
GSH	V plasma	0.14 (0.10, 0.41)	0.14 (0.09, 0.28)	0.014 (−0.047 to 0.067)	0.629
GSH	A blood cells	460 ± 87	547 ± 111	87.1 (3.8 to 170.4)	0.042
GSH	A plasma	0.49 ± 0.89	0.48 ± 0.91	0.005 (−0.035 to 0.025)	0.721
GSSG/GSH	V blood cells	1.2 ± 0.4	1.0 ± 0.4	−0.22 (−0.49 to 0.06)	0.113
GSSG/GSH	V plasma	0.07 (0.02, 0.64)	0.02 (0.01, 0.18)	−0.05 (−0.338 to −0.002)	0.013
GSSG/GSH	A blood cells	1.4 ± 0.5	1.3 ± 0.5	−0.06 (−0.41 to 0.28)	0.702
GSSG/GSH	A plasma	0.05 (0.01, 0.21)	0.09 (0.02, 0.22)	0.002 (−0.057 to 0.026)	0.624
GSSG	PA blood cells	670 ± 127	640 ± 108	−29.4 (−100.0 to 41.2)	0.387
GSSG	PA plasma	0.017 (0.002, 0.106)	0.007 (0.002, 0.103)	−0.006 (−0.024 to −0.001)	0.010
GSSG	V blood cells	189 ± 84	221 ± 127	31.6 (−41.0 to 104.1)	0.366
GSSG	V plasma	0.018 (0.003, 0.176)	0.005 (0.001, 0.48)	−0.002 (−0.218 to 0)	0.073
GSSG	A blood cells	622 ± 197	703 ± 269	80.8 (−80.8 to 242.5)	0.302
GSSG	A plasma	0.004 (0.001, 0.026)	0.011 (0.002, 0.055)	0.001 (−0.005 to 0.005)	0.480
$NADP^+$	PA blood cells	119 ± 20	179 ± 33	60.5 (39.6 to 81.3)	<0.0001
$NADP^+$	PA plasma	0.07 (0.01, 0.17)	0.03 (0.01, 0.08)	−0.007 (−0.082 to 0.005)	0.170
$NADP^+$	V blood cells	54 ± 24	89 ± 39	34.6 (8.8 to 60.4)	0.012
$NADP^+$	V plasma	0.05 (0.03, 0.12)	0.04 (0.03, 0.18)	0.005 (−0.056 to 0.019)	0.724
$NADP^+$	A blood cells	341 ± 88	361 ± 106	20.0 (−47.1 to 86.1)	0.539
$NADP^+$	A plasma	0.06 (0.03, 0.07)	0.07 (0.05, 0.16)	0.026 (−0.023 to 0.088)	0.307
NADPH	PA blood cells	2.8 ± 1.1	2.3 ± 1.2	−0.47 (−1.05 to 0.12)	0.111
NADPH	PA plasma	0.002 ± 0.001	0.002 ± 0.002	0 (−0.001 to 0.001)	0.724
NADPH	V blood cells	0.23 (0.15, 0.60)	0.25 (0.11, 0.74)	0.030 (−0.112 to 0.151)	0.478
NADPH	V plasma	0.002 (0.001, 0.003)	0.002 (0.001, 0.002)	0 (−0.001 to 0)	0.667
NADPH	A blood cells	2.9 (2.1, 3.7)	2.0 (1.4, 2.6)	−1.00 (−2.33 to 0.42)	0.268
NADPH	A plasma	0.002 (0.001, 0.002)	0.002 (0, 0.003)	0 (−0.001 to 0.002)	0.894

$NADP^+/NADPH$	PA blood cells	40 (33, 58)	80 (57, 102)	32.0 (15.0 to 42.8)	0.003
$NADP^+/NADPH$	PA plasma	66 ± 87	55 ± 85	−11.3 (−37.7 to 23.9)	0.548
$NADP^+/NADPH$	V blood cells	216 (107, 368)	201 (101, 518)	17.3 (−20.9 to 46.4)	0.443
$NADP^+/NADPH$	V plasma	98 ± 157	80 ± 120	−18.5 (−73.4 to 36.3)	0.473
$NADP^+/NADPH$	A blood cells	115 (74, 152)	161 (139, 260)	56.2 (3.3 to 159.8)	0.132
$NADP^+/NADPH$	A plasma	61 ± 82	159 ± 232	97.4 (−79.2 to 274.6)	0.249
NAD^+	PA blood cells	3.0 ± 2.0	3.5 ± 4.4	0.57 (−1.32 to 2.45)	0.530
NAD^+	PA plasma	0.01 (0, 0.12)	0.01 (0.01, 0.04)	0.001 (−0.030 to 1.371)	0.679
NAD^+	V blood cells	0.95 ± 0.96	1.52 ± 1.69	0.56 (−0.40 to 1.52)	0.227
NAD^+	V plasma	0.01 (0.01, 0.11)	0.02 (0, 0.13)	−0.006 (−0.028 to 0.002)	0.182
NAD^+	A blood cells	2.0 ± 2.4	4.1 ± 5.6	2.2 (−0.95 to 5.27)	0.158
NAD^+	A plasma	0.013 (0.005, 0.098)	0.017 (0.008, 0.067)	−0.004 (−0.035 to 0.003)	0.167
NADH	PA blood cells	1.5 (1.2, 2.4)	2.2 (1.6, 3.3)	0.55 (0.13 to 0.97)	0.025
NADH	PA plasma	0.003 (0.001, 0.007)	0.003 (0.002, 0.004)	0 (−0.002 to 0.001)	0.906
NADH	V blood cells	1.31 ± 0.89	1.54 ± 1.42	0.23 (−0.66 to 1.09)	0.587
NADH	V plasma	0.003 (0.002, 0.005)	0.003 (0.002, 0.004)	−0.001 (−0.005 to 0.002)	0.196
NADH	A blood cells	4.4 ± 3.2	4.4 ± 3.3	−0.02 (−1.40 to 1.36)	0.972
NADH	A plasma	0.004 ± 0.004	0.004 ± 0.004	0 (−0.004 to 0.003)	0.782
$NAD^+/NADH$	PA blood cells	1.49 ± 0.69	1.18 ± 1.03	−0.31 (−0.78 to 0.16)	0.183
$NAD^+/NADH$	PA plasma	2.5 (1.9, 5.5)	2.7 (1.5, 5.2)	0.36 (−3.38 to 1.98)	0.724
$NAD^+/NADH$	V blood cells	0.41 (0.29, 1.40)	0.68 (0.28, 1.32)	0.010 (−0.160 to 0.618)	0.712
$NAD^+/NADH$	V plasma	6.3 ± 7.8	4.9 ± 5.1	−1.3 (−4.55 to 1.87)	0.378
$NAD^+/NADH$	A blood cells	0.10 (0.09, 0.75)	0.69 (0.08, 1.27)	0.258 (−0.017 to 1.134)	0.105
$NAD^+/NADH$	A plasma	4.9 ± 5.0	5.1 ± 4.4	0.23 (−2.36 to 2.92)	0.845
Arg	PA blood cells	3.82 ± 2.13	3.14 ± 1.63	−0.68 (−1.52 to 0.16)	0.103
Arg	PA plasma	36 ± 23	39 ± 22	2.72 (−4.19 to 9.63)	0.413
Arg	V blood cells	0.99 (0.87, 1.72)	0.84 (0.64, 1.40)	−0.082 (−0.281 to 0.248)	0.712
Arg	V plasma	23 (15, 40)	32 (22, 42)	9.56 (0.46 to 11.26)	0.011
Arg	A blood cells	2.6 (2.1, 3.5)	3.9 (2.2, 5.2)	0.44 (−0.14 to 1.31)	0.244
Arg	A plasma	41 ± 29	37 ± 28	−3.67 (−10.88 to 3.55)	0.292
Cys	PA plasma	0.24 (0.13, 0.34)	0.28 (0.17, 0.32)	0.045 (−0.022 to 2.269)	0.083
Cys	V plasma	0.19 (0.15, 0.65)	0.28 (0.14, 0.38)	0.058 (0.002 to 1.344)	0.021

Cys	A plasma	0.21 (0.19, 0.39)	0.25 (0.14, 0.38)	−0.041 (−0.112 to 0.019)	0.103
VC	PA blood cells	0.47 ± 0.26	0.36 ± 0.19	−0.11 (−0.25 to 0.01)	0.095
VC	PA plasma	1.32 (1.06, 4.76)	1.08 (0.49, 5.24)	0.013 (−0.085 to 1.215)	0.477
VC	V blood cells	0.20 ± 0.15	0.11 ± 0.13	−0.082 (−0.182 to 0.018)	0.101
VC	V plasma	1.6 (0.2, 9.3)	0.7 (0.2, 5.1)	0.032 (−0.109 to 1.057)	0.712
VC	A blood cells	0.30 (0.25, 0.41)	0.36 (0.24, 0.49)	0.008 (−0.079 to 0.116)	0.514
VC	A plasma	1.3 ± 1.1	1.5 ± 1.3	0.140 (−0.394 to 0.674)	0.573

Redox biomarkers are reported as analyte-to-internal-standard peak-area ratios normalized to the actual sample volume used for extraction and are expressed as volume-normalized relative response units rather than absolute concentrations. All analyses were exploratory. *P* values are nominal and were not adjusted for multiple comparisons.

$NADP^+$, nicotinamide adenine dinucleotide phosphate; NADPH, reduced nicotinamide adenine dinucleotide phosphate; NAD^+, nicotinamide adenine dinucleotide; NADH, reduced nicotinamide adenine dinucleotide; Arg, arginine; Cys, cysteine; VC, vitamin C (ascorbic acid); PA, pulmonary arterial; V, peripheral venous; A, peripheral arterial; RIC, remote ischemic conditioning.

Table S3. Hemodynamic and Oxygen-Transport Variables Before and After Remote Ischemic Conditioning

Variables	Pre-RIC (N=15)	Post-RIC (N=15)	Mean/Median Difference	Nominal *P*
**Oxygen Transport**				
$AaDO_2$ (mmHg)	63 ± 46	65 ± 40	1.3 (−4.6 to 7.1)	0.653
CaO_2 (mL/dL)	17 (14–21)	16 (14–20)	−0.1 (−0.2 to 0.2)	0.666
$CmvO_2$ (mL/dL)	12 (10–14)	12 (9–14)	−0.2 (−0.5 to 0.2)	0.265
$Ca-mvO_2$ (mL/dL)	5.49 ± 1.48	5.50 ± 1.79	0.06 (−0.56 to 0.69)	0.828
DO_2 (mL/min)	724 (655–788)	670 (603–760)	−30.2 (−69.8 to 9.4)	0.123
VO_2 (mL/min)	236 ± 61	217 ± 49	−10.8 (−37.4 to 15.8)	0.396
O_2ER	0.32 ± 0.08	0.33 ± 0.10	0 (−0.03 to 0.04)	0.804
Qs/Qt (%)	17 (14–21)	18 (15–22)	−0.7 (−2.8 to 2.8)	1.000
**Hemodynamic**				
HR (bpm)	84 ± 17	80 ± 15	−2.6 (−6.2 to 1.0)	0.138
BP (mmHg)	87 ± 12	91 ± 15	3.7 (−2.4 to 9.8)	0.219
mPAP (mmHg)	49.3 ± 12.7	48.7 ± 12.6	−0.6 (−3.1 to 1.9)	0.621
RAP (mmHg)	3.0 (2.0–7.5)	2.0 (1.5–8.0)	0 (−0.5 to 0.5)	0.454
RVP (mmHg)	32 (22–37)	29 (22–35)	−1.0 (−4.0 to 0.0)	0.489
PCWP (mmHg)	8.0 (6.5–10.0)	8.0 (7.0–9.5)	0 (0 to 0)	0.752
CO (L/min)	4.4 ± 0.9	4.2 ± 0.9	−0.17 (−0.42 to 0.08)	0.158
CI ($L/min/m^2$)	2.7 ± 0.5	2.6 ± 0.6	−0.10 (−0.25 to 0.06)	0.206
SV (mL)	55 ± 17	55 ± 18	−0.5 (−4.5 to 3.6)	0.812
PVR ($dyn.sec/cm^5$)	793 ± 313	826 ± 356	33.5 (−36.5 to 103.6)	0.322
SVR ($dyn.sec/cm^5$)	1539 ± 482	1675 ± 499	136.3 (−13.3 to 285.9)	0.071

Continuous variables are presented as mean ± standard deviation or median (interquartile range), as appropriate. Paired differences were calculated as post-RIC minus pre-RIC values. All analyses were exploratory. P values are nominal and were not adjusted for multiple comparisons. Abbreviations: $AaDO_2$, alveolar–arterial oxygen gradient; CaO_2, arterial oxygen content; $CmvO_2$, mixed venous oxygen content; $Ca-mvO_2$, arteriovenous oxygen content difference; DO_2, oxygen delivery; VO_2, oxygen consumption; O_2ER, oxygen extraction ratio;

Qs/Qt, shunt fraction; HR, heart rate; BP, blood pressure; mPAP, mean pulmonary arterial pressure; RAP, right atrial pressure; RVP, mean right ventricular pressure; PCWP, pulmonary capillary wedge pressure; CO, cardiac output; CI, cardiac index; SV, stroke volume; PVR, pulmonary vascular resistance; SVR, systemic vascular resistance.
